# LINC00309 is associated with short disease-free survival in breast cancer

**DOI:** 10.1186/s12935-019-0887-x

**Published:** 2019-08-07

**Authors:** Sheng Huang, Yayun Chi, Weiru Chi, Rong Guo, Yonghui Su, Jingyan Xue, Shaoqiang Zhou, Jiankui Wang, Zhuangqing Yang, Jianyun Nie, Zhimin Shao, Dedian Chen, Jiong Wu

**Affiliations:** 1 Department of Breast Surgery, Key Laboratory of Breast Cancer in Shanghai, Fudan University Shanghai Cancer Center, Building 2, No. 270 Dong An Road, Shanghai, 200032 China; 2 Department of Oncology, Shanghai Medical College, Fudan University, Shanghai, China; 3 The 2nd Department of Breast Surgery, Breast Cancer Center of the Third Affiliated Hospital of Kunming Medical University, Tumor Hospital of Yunnan Province, Building 3, No. 519 Kunzhou Road, Kunming, 650118 China

**Keywords:** Breast cancer, Endocrine therapy resistance, Long non-coding RNA, LINC00309

## Abstract

**Background:**

Long non-coding RNAs play an important role in breast cancer. Even with adjuvant hormone therapy, patients with estrogen receptor positive breast cancer can present with recurrences and distant metastases. We investigated whether the expression of a novel long non-coding RNA LINC00309 can predict the outcome of breast cancer, especially for hormone-receptor positive patients.

**Methods:**

This retrospective study collected 290 breast cancer patients including 161 patients with hormone-positive. qPCR was performed to detect the expression of LINC00309. Kaplan–Meier and Cox risk proportion model were applied to disclose the function of LINC00309 for breast cancer prognosis.

**Results:**

LINC00309 high expression was an independent predictor for worse disease-free survival (*HR* = 2.127; 95% CI 1.074–4.212; *p* = 0.030) and associated with a shorter disease-free survival (*p* = 0.027), especially in hormone-positive breast cancer patients (*p *= 0.001). Also LINC00309 high expression was associated with a shorter disease-free survival both in selective estrogen receptor modulator related hormone therapy (*p *= 0.025) and aromatase inhibitors related hormone therapy (*p *= 0.048). Moreover, LINC00309 was an independent predictor of worse disease-free survival in hormone-receptor positive breast cancer patients on univariate (*HR* = 4.505; 95% CI 1.722–11.785; *p* = 0.002) and multivariate (*HR* = 4.159; 95% CI 1.537–11.251; *p* = 0.005) analyses.

**Conclusion:**

In breast cancer, Linc00309 is significantly associated with poor prognosis and may represent a new marker of prognosis.

**Electronic supplementary material:**

The online version of this article (10.1186/s12935-019-0887-x) contains supplementary material, which is available to authorized users.

## Background

Breast cancer (BC) is the most common malignant tumor among women worldwide [1]. Hormone-receptor (HR) positive BC is the largest therapeutic subtype of the disease, accounting for 60% to 75% of all the kinds’ of the malignant neoplasm breast disease [2]. For more than 50 years, the treatment of HR positive BC has been focused on targeting the estrogen receptor (ER) signaling pathway. Overcoming primary or secondary endocrine resistance in BC remains critical to further enhance the benefit of existing therapies, such as tamoxifen or aromatase inhibitors (AIs) or fulvestrant.

In humankind, approximately 19,000 protein-coding genes (PCGs) have been found [3] counting less than 2% of the total genome [4], whereas up to 70% of the human genome is transcribed only into RNA, yielding many thousands of non-coding RNAs [5]. Long non-coding RNAs (lncRNAs) are generated through PCGs similar pathways, with similar histone-modification profiles, splicing signals, and exon/intron lengths [5]. They have key roles in diverse biological processes, and their interfacing with epigenetic regulatory pathways resulted in emerging scientific interest [6, 7]. In cancer, in addition to genetic mutations of tumor suppressors or oncogene, lncRNAs may mediate oncogenic or tumor suppressing effects and promise to be a new class of cancer therapeutic targets [8]. LncRNAs may also serve as cancer diagnostic or prognostic biomarkers. Elevated expression level of homeobox antisense intergenic RNA (HOTAIR), a 2.2-kb lncRNA, correlate with BC, and is linked to poor prognosis and metastasis [9]. Some other lncRNAs could be used to predict carcinoma phenotypes and survival [10]. For cancer diagnosis, a well-known example is prostate cancer antigen 3 (PCA3). Noninvasive monitoring of urinary PCA3 transcript levels is currently developed for clinical diagnostics [11].

LINC00309 is located on 2p14. It was supposed to be a potential driver of lncRNAs, associated with cancer genomic alterations and correlated with patient survival [12] in a study on 5037 human tumor specimens of 13 cancer types in The Cancer Genome Atlas database. Our study is the first study suggesting that LINC00309 may represent a predictive marker of endocrine therapy (ET) resistance for HR-positive BC.

## Methods

### Tissue samples and clinical data

Our study was approved by the clinical ethical committee of Fudan University Shanghai Cancer Center (FDUSCC, Shanghai, PR China). Human BC tissue samples were obtained from the Department of Breast Surgery in FDUSCC after obtaining informed consent from the patients diagnosed with BC. We confirmed that all methods were performed in accordance with the relevant guidelines and regulations. A total of 290 primary BC samples of stage I to III invasive ductal carcinoma cases (collected postoperatively from February 2007 to December 2012). Patients who received systemic therapy before sample collection or those diagnosed with metastases were excluded.

Clinicopathological features were mainly collected from medical records, pathology reports, and personal interviews, including baseline of patients, surgery information, pathological data, and follow-up data of the tumor. Clinical staging criteria were assessed according to the American Joint Committee (2010) on Cancer TNM classification. The pathological diagnosis; expression status of ER, progesterone receptor (PR), and human epidermal growth factor 2 (HER-2); and Ki67 status were determined by at least two academic pathologists according to the World Health Organization (WHO) classification and American Society for Clinical Oncology (ASCO) guidelines.

Therapeutic regimen decisions were based on the Chinese Anti-Cancer Association guidelines for BC diagnosis and treatment. In this study, we defined the selective ER modulator (SERM)-related hormone therapy as tamoxifen only, tamoxifen plus luteinizing hormone releasing hormone (LHRH), and raloxifene-related (including raloxifene only and raloxifene mainly strategies). The AIs-related ET was defined as AIs only, AIs plus LHRH, and AIs followed by fulvestrant. Patients who received tamoxifen followed by the AIs regimen were classified according to the time rule, namely they were included in the tamoxifen group if the duration of tamoxifen therapy was longer than that of AIs regimen; otherwise, patients were included in the AIs group. In addition, patients who switched from primary ET to another therapy because of the former’s side effects also followed the time rule. The proportion of patients receiving the different therapeutic regimens is shown in Table 2.

### Lab experiments

Total RNA was extracted from tissues using the TRIzol reagent (Invitrogen, Carlsbad, CA, USA). real-time quantitative polymerase chain reaction (RT-qPCR) was performed using SYBR Premix Ex Taq kit (Takara Bio Inc., Otsu, Japan) and ABI 7900 system (Applied Biosystems, Foster City, CA, USA) as previously described [13]. Relative expression of LINC00309 was calculated with GAPDH using the comparative delta–delta CT method (2-delta Ct). All reactions were performed in triplicate. The primers sequences were as follows: LINC00309 forward: 5′-GCCCCTAGGGAGAAATGCAG-3′; LINC00309 reverse: 5′-GGCCAGTGCTCTTCTGAACT-3′.

### Statistical and bioinformatics analysis

The interval from the date of initial surgery to disease progression (the first recurrence of disease at a local, regional, or distant site; the diagnosis of contralateral BC; and breast-cancer-specific death) was defined as disease free survival (DFS). Patients lost to follow-up at the study end date were censored. The best sensitivity and specificity point of receiver operating characteristic (ROC) DFS curves were used to define LINC00309 with low or high level expression. Correlations between the clinicopathological parameters and markers of interest were evaluated using contingency tables and Pearson’s χ^2^ or Fisher’s exact tests. The postoperative DFS probability was derived from the Kaplan–Meier (KM) estimate and compared using the log-rank test. Univariate and multivariate analyses were performed using the Cox risk proportion model. Statistical analyses were performed using SPSS 21.0 software (SPSS Inc., Chicago, IL, USA). All p-values were two-sided, and p-values less than 0.05 were considered statistically significant. All analyses were based on the observed data with the assumption that missing data were random.

## Results

### The predictive role of LINC00309 expression for BC patients prognosis

**Fig. 1:**
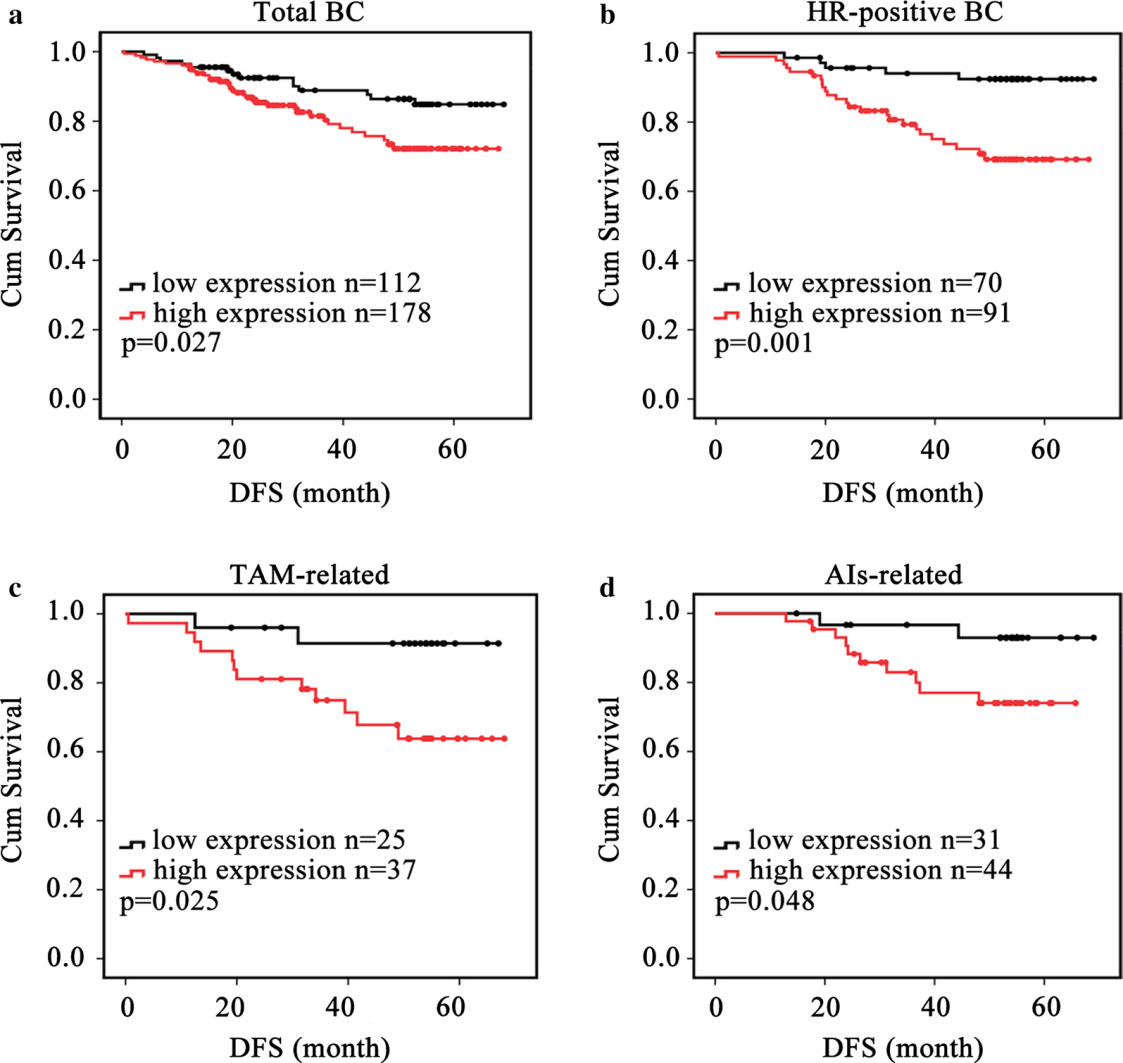
Kaplan–Meier survival curves of breast cancer patients based on LINC00309 expression status. (black lines indicate patients with low LINC00309 expression; red lines indicate patients with high LINC00309 expression). **a** Cumulative disease free survival curves of 290 breast cancer patients with high or low LINC00309 expression (*p* = 0.027). **b** Cumulative disease-free survival curves of 161 HR-positive breast cancer patients with high or low LINC00309 expression (*p* = 0.001). **c** Cumulative disease-free survival curves according to LINC00309 expression status of 62 HR-positive breast cancer patients who received primary SERM-related therapy (*p* = 0.025). **d** Cumulative disease-free survival curves according to LINC00309 expression status of 75 HR-positive breast cancer patients who received primary aromatase inhibitor-related therapy (*p* = 0.048)

For the total 290 patients, after a mean follow-up time of 51 months, 50 patients experienced disease recurrence. According to DFS ROC curve, 112 cases were in LINC00309 low group, and the other 178 cases were in the LINC00309 high group. LINC00309 high expression was an independent poor predictor for DFS BC based on both univariate (*HR* = 1.990; 95% CI 1.069–3.703; *p* = 0.030) and adjusted multivariate survival analyses (*HR* = 2.127; 95% CI 1.074–4.212; *p* = 0.030) (Table 1). LINC00309 high expression also associated with poor DFS in BC upon the KM analysis (*p* = 0.027; Fig. 1a). KM analysis was then used to view LINC00309 expression and DFS relationship in all four molecular subtypes: Luminal A, Luminal B, HER-2 overexpression and basal-like BC for these 290 patients. The data showed that LINC00309 high expression associated with poor DFS mainly in Luminal A subtype (*p* = 0.001) (Additional file 1: Figure S1A), but not in the Luminal B (*p* = 0.073) (Additional file 1: Figure S1B), Her2 positive (*p* = 0.422) and basal-like BC (*p* = 0.471) (data not show). Then, the prognostic prediction of LINC00309 expression was analyzed in these four biomarkers separately in the total 290 patients. LINC00309 high expression could be a poor predictor for ki-67 negative (*p* = 0.016) (Additional file 1: Figure S1C), HER-2 negative (*p* = 0.011) (Additional file 1: Figure S1D), PR positive (*p* = 0.001) (Additional file 1: Figure S1E) and ER positive (*p* < 0.001) (Additional file 1: Figure S1F) patients. Base on these results, we put focus on the LINC00309 function in HR positive patients.

**Table 1:** Univariate and multivariate analysis for disease-free survival in total 290 cases

	Univariate analysis		Multivariate analysis	
	Hazard ratio (95% CI)	*p*^a^ value	Hazard ratio (95% CI)	*p*^a^ value
Age				
> 40 vs. ≤ 40	1.618 (0.828–3.159)	0.159	1.386 (0.695–2.762)	0.354
Tumor size				
≤ 2 vs. > 2	1.444 (0.865–2.410)	0.159	1.133 (0.651–1.9718)	0.434
Lymph node status				
Negative vs. positive	5.855 (1.045–1.518)	*0.016*	1.544 (1.102–1.529)	*0.002*
Vessel invasion				
Negative vs. positive	2.311 (1.304–4.097)	*0.004*	1.779 (0.816–2.924)	0.182
ER status				
Negative vs. positive	0.703 (0.396–1.250)	0.230	0.150 (0.023–0.974)	0.057
PR status				
Negative vs. positive	0.807 (0.453–1.438)	0.467	5.466 (0.831–35.955)	0.077
Ki-67				
Negative vs. positive	1.911 (1.075–3.395)	*0.027*	1.053 (1.053–3.733)	*0.034*
HER-2/neu status				
Negative vs. positive	0.712 (0.403–1.260)	0.244	0.324 (0.324–1.143)	0.123
LINC00309				
Low vs. high	1.990 (1.069–3.703)	*0.030*	2.127 (1.074–4.212)	*0.030*

*CI* confidence interval, *HER-2* human epidermal growth factor receptor 2, *PR* progesterone receptor

^a^*p* is based on the Cox regression test

### Relevance of LINC00309 expression and clinicopathological characteristics in HR-positive BC

In these 290 patients, 161 patients were HR-positive. 70 cases showed low LINC00309 expression and 91 cases showed high LINC00309 expression. For these HR-positive patients, 30 patients experienced disease recurrence after a mean follow-up of 53 months. To identify the clinical relevance of LINC00309 expression in HR-positive BC, the correlations between LINC00309 expression and clinicopathological parameters, such as age, histological grade, tumor size, lymph node status, vessel invasion, HER-2, and Ki-67, were examined (Table 2). In HR-positive BC, LINC00309 expression was significantly correlated with HER-2 negative (*p* = 0.011). However, LINC00309 expression in BC was not associated with other parameters (Table 2). The distribution of chemotherapeutic or ET regimens in the low and high LINC00309 expression groups showed no significant difference (Table 2).

**Table 2:** Clinicopathological variables and the expression of LINC00309 in HR-positive cases

Variables	Number of patients (%)	LINC00309 expression		*p*^a^ value
		Low n (%)	High n (%)	
Total	161	70 (43.5)	91 (56.5)	
Age				0.942
≤ 40 years	28 (17.4)	12 (7.5)	16 (9.9)	
> 40 years	133 (82.6)	58 (36.0)	75 (46.6)	
Tumor size				0.552
T1	59 (36.6)	23 (14.3)	36 (22.4)	
T2	96 (59.6)	45 (28.0)	51 (31.7)	
T3	6 (3.7)	2 (1.2)	4 (2.5)	
Lymph node status				0.824
pN0	69 (42.9)	31 (19.3)	38 (23.6)	
pN1	45 (28.0)	19 (11.8)	26 (16.1)	
pN2	21 (13.0)	9 (5.6)	12 (7.5)	
pN3	25 (15.5)	10 (6.2)	15 (9.3)	
Unknown	1 (0.6)	1 (0.6)	0 (0.0)	
Vessel invasion				0.708
Negative	87 (54.0)	39 (24.2)	48 (29.8)	
Positive	74 (46.0)	31 (19.3)	43 (26.7)	
HER-2/neu status				*0.011*
Negative	120 (74.5)	46 (28.6)	74 (46.0)	
Positive	36 (22.4)	19 (11.8)	17 (10.6)	
Unknown	5 (3.1)	5 (3.1)	0 (0.0)	
Ki-67 status				0.489
Negative	108 (67.1)	49 (30.4)	59 (36.6)	
Positive	53 (32.9)	21 (13.0)	32 (19.9)	
Chemotherapy regimes				0.271
CAF^b^	30 (18.6)	15 (9.3)	15 (9.3)	
TC (± H)^c^	11 (6.8)	2 (1.2)	9 (5.6)	
CAF-T (± H)^d^	85 (52.8)	39 (24.2)	46 (28.6)	
Other	6 (3.7)	4 (2.5)	2 (1.2)	
None	20 (12.4)	6 (3.7)	14 (8.7)	
Unknown	9 (5.6)	4 (2.5)	5 (3.1)	
Hormone therapy				0.358
SERMs related	62 (38.5)	25 (15.5)	37 (23.0)	
TAM only	38 (23.6)	14 (8.7)	24 (14.9)	
TAM + LHRH	6 (3.7)	1 (0.6)	5 (3.1)	
TAM (major)-AIs^e^	6 (3.7)	3 (1.9)	3 (1.9)	
Others^f^	12 (7.5)	7 (4.3)	5 (3.1)	
AIs related	75 (46.6)	31 (19.3)	44 (27.3)	
AIs only	60 (37.3)	25 (15.5)	35 (21.7)	
AIs + LHRH	3 (1.9)	1 (0.6)	2 (1.2)	
TAM-AIs (major)^g^	9 (5.6)	5 (3.1)	4 (2.5)	
AIs-others^h^	3 (1.9)	0 (0.0)	3 (1.9)	
Unknown	24 (14.9)	14 (8.7)	10 (6.2)	

*HR* hormone-receptor, *HER-2* human epidermal growth factor receptor 2, *SERMs* selective estrogen receptor modulators, *TAM* tamoxifen, *LHRH* luteinizing hormone releasing hormone, *AIs* aromatase inhibitors

^a^*p* is based on Pearson’s χ2 or Fisher’s exact tests

^b^Anthracycline plus cyclophosphamide and 5-fluorouracil

^c^Taxanes plus cyclophosphamide combined with herceptin or not

^d^Anthracycline-based chemotherapy followed by taxanes combined with herceptin or not

^e^The time patient took tamoxifen longer than the time to take AIs in tamoxifen followed by AIs regime

^f^Raloxifene related, including raloxifene only and raloxifene mainly strategies

^g^The time patient took tamoxifen shorter than the time to take AIs in tamoxifen followed by AIs regime

^h^The time patient took AIs longer than the time to take fulvestrant in AIs followed by fulvestrant regime

### Elevated LINC00309 expression is associated with poor disease-free survival in HR-positive BC

To assess the clinical significance of LINC00309 overexpression, we analyzed the relationship between LINC00309 expression and DFS in HR-positive BC. Both univariate and adjusted multivariate survival analyses revealed a significant difference between the LINC00309 high and low expression groups. Patients with high LINC00309 expression had a higher likelihood for disease events in univariate analysis (*HR* = 4.505; 95% CI 1.722–11.785; *p *= 0.002) and a similar trend in multivariate analysis (*HR* = 4.159; 95% CI 1.537–11.251; *p *= 0.005) (Table 3). Additionally, patients with high LINC00309 generally demonstrated poor DFS in HR-positive BC upon KM analysis (*p *= 0.001) (Fig. 1b). Furthermore, even in patients with different ET regimens, high LINC00309 expression was associated with the same results. For patients who received SERM-related ET, high LINC00309 expression was also associated with a poor DFS (*p *= 0.025) (Fig. 1c). The same results were found in patients treated with AIs-related ET (*p *= 0.048) (Fig. 1d). Thus, these results strongly indicate that high LINC00309 expression is directly associated with recurrent disease in patients with HR-positive BC, regardless of the received ET regimens.

**Table 3:** Univariate and multivariate analysis for disease-free survival in 161 HR-positive cases

	Univariate analysis		Multivariate analysis	
	Hazard ratio (95% CI)	*p*^a^ value	Hazard ratio (95% CI)	*p*^a^ value
Age				
> 40 vs. ≤ 40	2.312 (1.058–5.054)	*0.036*	1.532 (0.677–3.465)	0.306
Tumor size				
≤ 2 vs. > 2	1.535 (0.791–2.978)	0.205	1.149 (0.567–2.327)	0.700
Lymph node status				
Negative vs. positive	1.236 (1.022–1.495)	*0.029*	1.141 (0.840–1.549)	0.398
Vessel invasion				
Negative vs. positive	2.267 (1.078–4.767)	*0.031*	1.899 (0.790–4.563)	0.151
Ki-67				
Negative vs. positive	2.230 (1.087–4.575)	*0.029*	2.478 (1.144–5.368)	*0.021*
HER-2/neu status				
Negative vs. positive	0.384 (0.141–1.046)	0.061	0.453 (0.160–1.286)	0.137
LINC00309				
Low vs. high	4.505 (1.722–11.785)	*0.002*	4.159 (1.537–11.251)	*0.005*

*HR* hormone-receptor, *CI* confidence interval, *HER-2* human epidermal growth factor receptor 2

^a^*p* is based on the Cox regression test

## Discussion

LncRNAs were divided into 5 categories and lincRNA, showing intergenic non-coding RNA loci with a length > 200 bp, is one of them [5]. lncRNAs can be located in the nucleus and the cytoplasm. In the nucleus, it could have an intrinsic role in enhancer function [14], establishment or maintenance of chromosome conformation and organization of nuclear architecture [15] or regulation of alternative splicing [16]. In the cytoplasm, lncRNAs can modulate mRNA stability and translation, and sequester microRNAs (miRNAs) by functioning as decoys (miRNA sponges) [17]. LncRNAs can also be localized in other subcellular compartments, such as ribosomes and mitochondria [18]. To date, three main drug types are used for hormone-dependent BC treatment: SERMs, such as tamoxifen, which antagonizes the ER at the nuclear level [19]; selective ER downregulators (SERDs), such as fulvestrant, which induce destabilization and degradation of ER; and AIs, which reduce estrogen production in the peripheral tissues and within the tumors through inhibition of the aromatase enzyme [20]. The first-line ET therapies for hormone-dependent BC with no metastatic loci are SERMs and AIs [20], and the same strategy was used in this study. However, a significant number of hormone-dependent patients failed to respond to ET because of resistance. Although SERMs, SERDs, and AIs may be involved in different mechanisms of ER down-regulation in BC cells, the core mechanisms that contribute to ET resistance are estrogen hypersensitivity, ER changes (i.e., receptor loss, mutations, or gene expression changes), intracellular environmental molecular changes (i.e., PR loss, changes in the expression of cofactors), and increased molecular cross-talking between ER and growth factor receptor signaling pathways [21] leading to the dysregulation of PI3K-PTEN/AKT/mTOR, RAS/MEK/MAPK, and NF-κB pathways [21]. Two isoforms of ERs, ER-α and ER-β, are known [22]. ERα plays a crucial role in BC initiation and progression [22, 23]. Additionally, abnormal metabolism of the drug leads to ET resistance [24].

Some studies, like growth arrest-specific transcript 5 (GAS5) [25], HOTAIR [9], and BC anti-estrogen resistance 4 (BCAR4) [26], examined the relationship between lncRNA and BC ET resistance. Low GAS5 expression was found in BC samples and it was considered a distinct tumor suppressor that prevented the glucocorticoid receptor from binding to the target DNA. Interestingly, GAS5 interacted with the androgen receptor and progesterone receptor, but not with ER [25]. HOTAIR overexpression could enhance metastasis and invasion of BC cells, leading to poor overall survival and progression-free survival [9]. The underlying mechanism is that HOTAIR regulates the homeobox D (HOXD) cluster by tethering the polycomb repressor complex 2 (PRC2) protein to the DNA at this site. PRC2 is able to promote histone H3K27 trimethylation and subsequent repression of transcription at the HOXD cluster, thereby preventing differentiation and leading to an invasive cellular phenotype [27]. BCAR4 is a clinical biomarker for increased invasiveness and tamoxifen resistance in BC [26, 28]. The role of BCAR4 in tamoxifen resistance relies on the presence of HER2 and ERBB3 receptors [26], but is independent of ERα [28, 29]. A HER2 inhibitor may thus be ideal for patients whose tumors are resistant to traditional endocrine therapy due to high levels of BCAR4 [28]. Additionally, BCAR4 has tissue-specific expression, and is expressed only in BC cells, human placenta, and oocytes. This makes BCAR4 a good target for anti-estrogen resistance BC treatment [28]. About LINC00309, it was found by a bio-information research based on TCGA database [12]. But in this study, no further research focused on LINC00309 function. Another study further found LINC00309 with other two gene dernicidin (DCD1) and Chronic lymphocytic leukemia up-regulated 1 (CLLU1) were specific expression in tumor [30].

No other reports about the biological function of LINC00309 are available. Based on previous lncRNAs’ function and BC ET mechanism, we speculate that LINC00309 enhanced ET resistance through the direct influence on the up- or down-regulation of ER transcript-related gene expression function, which promotes BC cell growth and proliferation, or its influence on PI3K/AKT, mTOR, or NF-κB signal pathways. Further studies are necessary to investigate how LINC00309 influences ET resistance in BC.

## Conclusions

Our studies first uncover the role of a new lncRNA LINC00309 in breast cancer through investigating its expression in a cohort of breast cancer patients and analyzing its correlation with prognosis. Patients with elevated LINC00309 expression had a higher likelihood for disease events, especially in hormone-receptor positive patients either treated with SERM-related or AIs-related endocrine treatment. This suggested that LINC00309 could be a new marker of prognosis in breast cancer.

## Additional file

**Additional file 1: Figure S1.** Kaplan–Meier survival curves of molecular subtype breast cancer patients based on LINC00309 expression status. (black lines indicate patients with low LINC00309 expression; red lines indicate patients with high LINC00309 expression). A) Cumulative disease free survival curves of 85 Luminal A subtype with high or low LINC00309 expression (*p* = 0.001). B) Cumulative disease free survival curves of 75 Luminal B subtype with high or low LINC00309 expression (*p* = 0.073). C) Cumulative disease free survival curves of 132 ki-67 negative patients with high or low LINC00309 expression (*p* = 0.016). D) Cumulative disease free survival curves of 184 HER-2 negative with high or low LINC00309 expression (*p* = 0.011). E) Cumulative disease free survival curves of 157 PR-positive with high or low LINC00309 expression (p = 0.001). F) Cumulative disease free survival curves of 160 ER-positive with high or low LINC00309 expression (*p* < 0.001).

## Data Availability

Not applicable.

## Abbreviations

RNA: ribonucleic acid
RT-qPCR: real-time quantitative polymerase chain reaction
*HR*: hazard ratio
CI: confidence interval
HR: hormone-receptor
BC: positive breast cancer
AIs: aromatase inhibitors
PCGs: protein-coding genes
lncRNAs: long non-coding RNAs
HOTAIR: homeobox antisense intergenic RNA
PCA3: prostate cancer antigen 3
ET: endocrine therapy
PR: progesterone receptor
HER-2: human epidermal growth factor 2
WHO: World Health Organization
ASCO: American Society for Clinical Oncology
SERM: selective ER modulator
LHRH: luteinizing hormone releasing hormone
DFS: disease free survival
ROC: receiver operating characteristic
KM: Kaplan–Meier
miRNAs: microRNAs
SERDs: selective ER downregulators
PI3K: phosphatidylinositol 3-kinase
PTEN: phosphatase and tensin homolog
AKT: protein kinase B
mTOR: the mammalian target of rapamycin
MEK: mitogen-activated protein kinase kinase 7
MAPK: mitogen-activated protein kinase 1
NF-κB: nuclear factor-kappa B
GAS5: growth arrest-specific transcript 5
BCAR4: BC anti-estrogen resistance 4
HOXD: HOTAIR regulates the homeobox D
PRC2: polycomb repressor complex 2
