# Supplementary figures and images for: LINC00309 is associated with short disease-free survival in breast cancer
Source: Cancer Cell Int. 2019 Aug 7;19:210. doi: 10.1186/s12935-019-0887-x (PMC6686222; doi:10.1186/s12935-019-0887-x)

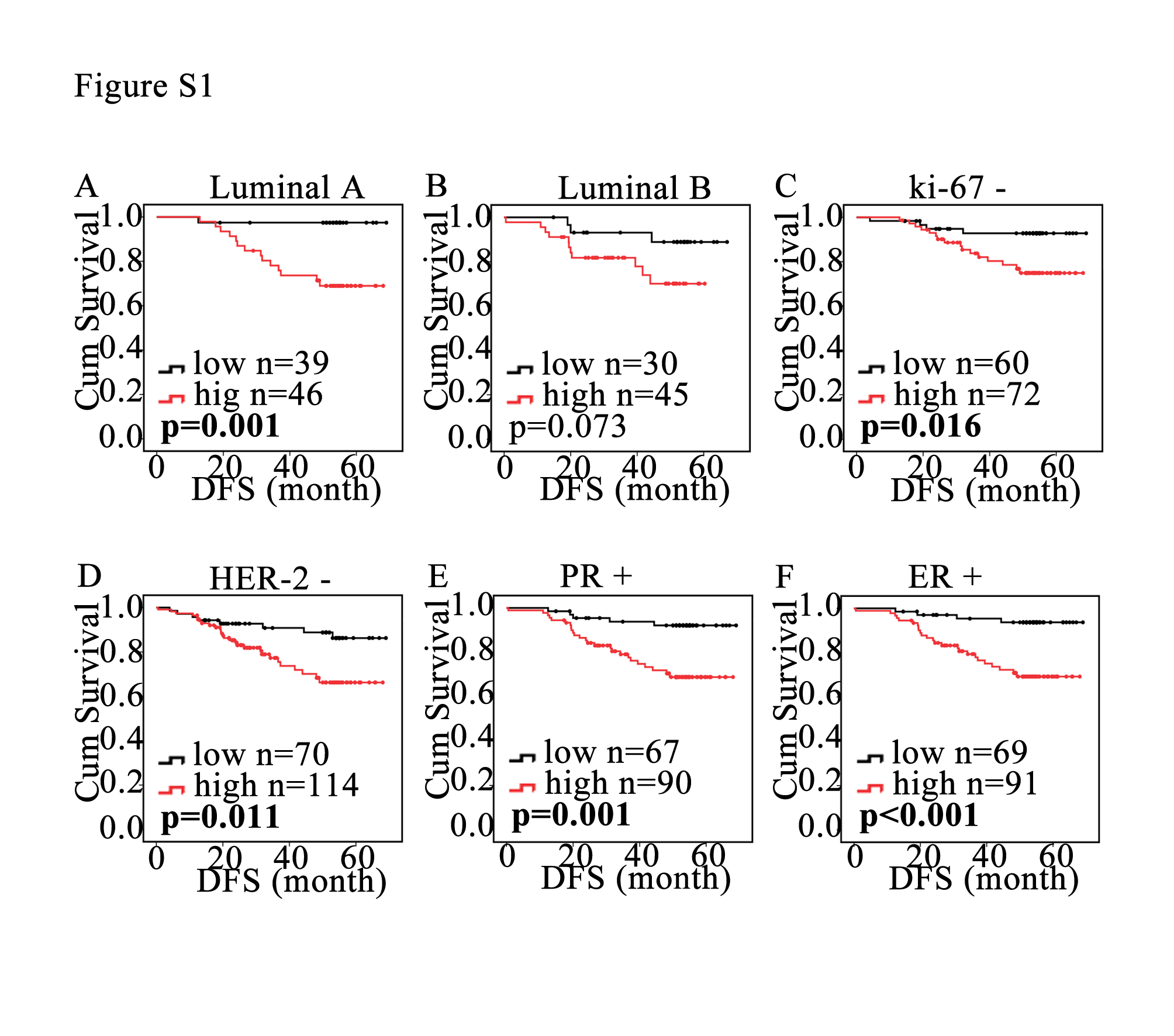

Supplement: Supplementary file 1 — Additional file 1: Figure S1. Kaplan–Meier survival curves of molecular subtype breast cancer patients based on LINC00309 expression status. (black lines indicate patients with low LINC00309 expression; red lines indicate patients with high LINC00309 expression). A) Cumulative disease free survival curves of 85 Luminal A subtype with high or low LINC00309 expression (p = 0.001). B) Cumulative disease free survival curves of 75 Luminal B subtype with high or low LINC00309 expression (p = 0.073). C) Cumulative disease free survival curves of 132 ki-67 negative patients with high or low LINC00309 expression (p = 0.016). D) Cumulative disease free survival curves of 184 HER-2 negative with high or low LINC00309 expression (p = 0.011). E) Cumulative disease free survival curves of 157 PR-positive with high or low LINC00309 expression (p = 0.001). F) Cumulative disease free survival curves of 160 ER-positive with high or low LINC00309 expression (p < 0.001). [file 12935_2019_887_MOESM1_ESM.png]
